# *Anopheles *mortality is both age- and *Plasmodium*-density dependent: implications for malaria transmission

**DOI:** 10.1186/1475-2875-8-228

**Published:** 2009-10-12

**Authors:** Emma J Dawes, Thomas S Churcher, Shijie Zhuang, Robert E Sinden, María-Gloria Basáñez

**Affiliations:** 1 Department of Infectious Disease Epidemiology, Faculty of Medicine, Imperial College London, London, UK; 2 Division of Cell and Molecular Biology, Faculty of Life Sciences, Imperial College London, London, UK

## Abstract

**Background:**

Daily mortality is an important determinant of a vector's ability to transmit pathogens. Original simplifying assumptions in malaria transmission models presume vector mortality is independent of age, infection status and parasite load. Previous studies illustrate conflicting evidence as to the importance of *Plasmodium*-induced vector mortality, but very few studies to date have considered the effect of infection density on mosquito survival.

**Methods:**

A series of three experiments were conducted, each consisting of four cages of 400-1,000 *Anopheles stephensi *mosquitoes fed on blood infected with different *Plasmodium berghei *ookinete densities per microlitre of blood. Twice daily the numbers of dead mosquitoes in each group were recorded, and on alternate days a sample of live mosquitoes from each group were dissected to determine parasite density in both midgut and salivary glands.

**Results:**

Survival analyses indicate that mosquito mortality is both age- and infection intensity-dependent. Mosquitoes experienced an initially high, partly feeding-associated, mortality rate, which declined to a minimum before increasing with mosquito age and parasite intake. As a result, the life expectancy of a mosquito is shown to be dependent on both insect age and the density of *Plasmodium *infection.

**Conclusion:**

These results contribute to understanding in greater detail the processes that influence sporogony in the mosquito, indicate the impact that parasite density could have on malaria transmission dynamics, and have implications for the design, development, and evaluation of transmission-blocking strategies.

## Background

Daily mortality is the most important determinant of a mosquito's ability to transmit pathogens, influencing the probability to encounter infectious hosts, survive the extrinsic incubation period and transmit the infection [1]. The period necessary for the parasite to reach its infective stage within the vector often takes an appreciable portion of the vector's life-span and, therefore, only a small proportion actually survive long enough in nature to transmit the infection. As a result, the basic reproduction number (*R*_0_) of vector-borne infections is critically dependent on the life-span of the vector, and in particular on the infective life expectancy [2,3]. Small changes in the daily mortality rate can result in relatively large changes in transmission. In support of this, Macdonald's malaria models indicated that at equilibrium, the weakest link in the chain of transmission was the survivorship of the adult female *Anopheles *[1], providing a rationale for a DDT-focused, World Health Organization-coordinated eradication campaign that successfully eliminated malaria transmission among approximately 700 million people [4]. Therefore, understanding the determinants of mosquito survival can have important implications for the design and assessment of new malaria control strategies.

Original simplifying assumptions in malaria transmission models include that vector mortality is independent of and, therefore, unaffected by, mosquito age, infection status and parasite load [1,3,5-7]. This has resulted in estimates of the daily survival rate entering as constants in mathematical equations of epidemiological indices such as the vectorial capacity and the entomological inoculation rate, in models of population dynamics and in the assessment of control strategies. These assumptions have continued to permeate malaria transmission models despite conflicting evidence as to their validity.

The assumption of mosquito mortality being independent of age was first articulated by Macdonald, who reasoned that environmental insults, disease, and predation would kill mosquitoes before they died of old age [5]. Macdonald, therefore, based his mathematical treatment of survival on the factor *p*, the probability of a mosquito surviving from one day to the next. Some studies support this notion [8,9] whereas others have found evidence of mosquito senescence, particularly in laboratory populations [10-16]. Notably, Clements and Patterson [17] re-analysed published reports of mosquito mortality and concluded that many species exhibit age-dependent mortality, with most, but not all, consistent with the Gompertz model [18]. More recently, Styer *et al *[16] found that mortality was highly age-dependent in both sexes of *Aedes aegypti*, and that the age at which a mosquito first bites an infectious host is an important indicator of the probability of transmitting a pathogen.

Despite these studies clearly calling into question the assumption of no senescence in mosquito populations, the common operational assumption remains that insect vector mortality is independent of age, and this has been incorporated into many mathematical models [7,19-24]. The reluctance for this to change can primarily be ascribed to the fact that allowing mortality to be constant with age leads to the exponential model for the distribution of survival times, which has the significant advantage of mathematical simplicity and tractability, and reduces the number and complexity of variables that need to be considered. However, acceptance of this non-senescence assumption leads to the simplified view that the potential of mosquitoes to survive and transmit disease is constant regardless of their age, and it has been shown that quantitative models that assume non-senescence can produce results with substantial errors [17].

It has been argued that there will be strong selection pressure on *Plasmodium *not to reduce vector survival, as both partners benefit from high rates of survival and of blood-feeding; the mosquito to increase its reproductive success and the parasite to ensure its transmission [25]. However, investigations into the pathogenicity of malarial parasites in mosquitoes have not been conclusive, resulting in conflicting evidence as to whether malaria parasites are benign to their vectors. Laboratory studies are contradictory; some indicate that the survival rate of infected mosquitoes is not different from that of non-infected mosquitoes [26-31], whereas others indicate reduced survival [32-37]. Ferguson and Read [38] conducted a meta-analysis of 22 previously published laboratory studies, and concluded that overall, malaria parasites do reduce mosquito survival, but stated that these mortality effects were more likely to be detected in vector-parasite combinations not occurring naturally in the field and in studies of longer duration. Field studies which have explored parasite-induced vector mortality indirectly, have also yielded conflicting results; some supporting [39,40] and others not supporting [41] its operation.

It has also been suggested that malaria parasites may only be harmful to mosquitoes when parasite burdens are exceedingly high [28,35], which has been used to refute the existence of *Plasmodium*-induced mortality in nature, as most naturally infected mosquitoes carry, on average, only two to three oocysts of *Plasmodium falciparum *[42-45]. However, the absence of high oocyst burdens in population samples could also be due to the mortality of more heavily infected mosquitoes [40]. Very few studies to date have explicitly and systematically considered the effect of infection density on mosquito mortality, and those which have, have not reported consistent results. Whilst some authors suggest that mosquito survivorship is not negatively correlated with parasite density [29,32], others found that mosquito mortality increased with oocyst burden [35-37,46]. The review by Ferguson and Read [38] concluded that there is no relationship between mortality and mean oocyst burden in the five studies that reported oocyst burden, but suggested that sporozoite load may be the prime determinant of mosquito mortality as mortality differences only became apparent in studies of longer duration when sporozoites would be in the salivary glands.

In the context of the renewed global efforts to eliminate malaria, it has become increasingly important to obtain a better understanding of the component of the malaria life cycle taking place within the mosquito. Using the *Plasmodium berghei-Anopheles stephensi *experimental system, it has been rigorously demonstrated that parasite development during sporogony is density-dependent [47]. Using the same system, this paper investigates the validity of the original simplifying assumptions that mosquito mortality is independent of age, infection status, and infection density, which are commonly used in the formulation of mathematical models of malaria transmission. The ultimate aim is that of generating testable hypotheses that serve to prompt investigation of whether similar phenomena apply to any of the complex, numerous, and multifarious parasite-vector combinations that play a role in malaria transmission in the field.

## Methods

### Experimental design

**Figure 1:**
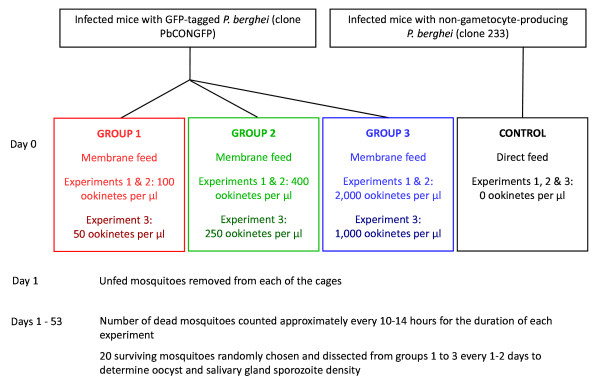
**Schematic representation of the experimental design**. Three experiments were conducted each consisting of 4 cages of *An. stephensi *mosquitoes, represented by boxes in the figure.

Three experiments were conducted over the course of one year, each consisting of four (30 cm^3^) cages of *An. stephensi *(SDA500 strain) fed on mouse blood infected with different *P. berghei *ookinete densities, summarized in Figure 1. The first group of mosquitoes in each experiment acted as the control group, and were fed on rodent blood containing *P. berghei *233; a non-gametocyte-producing clone (i.e., 0 ookinetes). This choice of control recognizes the impact of parasite-induced serum components present at the time of blood-feed which are known to modulate parasite infectivity [48], thus making the groups as comparable as possible, differing only in the presence and density of ookinetes. The further three groups were fed on blood containing increasing ookinete densities; 100, 400 and 2,000 ookinetes per μl of blood in the first two experiments (to represent the three phases of the sigmoid relationship between numbers of oocysts and ookinetes shown in Sinden *et al *[47]), and 50, 250 and 1,000 ookinetes per μl of blood in the third experiment (in order to explore a different range of parasite densities). Ookinete rather than gametocyte densities were chosen as the source of infection because they tend to predict more accurately the intensity of the resulting infection [47], and reduce the between-mosquito variability that would otherwise require much larger (and unfeasible) mosquito numbers to achieve sufficient statistical power. For these groups of mosquitoes the transgenic GFP-expressing *P. berghei *clone PbCONGFP (ANKA strain) was maintained in Theiler's Original mice, as these parasites express the GFP constitutively throughout all stages of the life cycle facilitating localization and enumeration of parasites. The growth kinetics of this fluorescent strain has been shown to be the same as that of the wild-type [49]. The course of infections and gametocyte production were monitored on Giemsa-stained blood films. The mosquitoes were starved overnight and fed either directly on anaesthetized infected mice (for the control cage), or were membrane-fed with a suspension of cultured ookinetes in blood from uninfected mice (for each of the other cages). The feeder apparatus used Parafilm^® ^as the feeding membrane and maintained the blood at a constant temperature of 37°C using a water circulation system. The feeds lasted approximately 90 minutes in the dark at 19°C. Those mosquitoes which had taken less than a full blood meal (distinguished visually) were removed the following day, reducing the possibility that any difference between groups could be due to variation in blood meal size, and resulting in roughly 400 to 1,000 fed females per cage (see Table 1). The cages were maintained at approximately 19°C, 80% relative humidity and fed on 5% fructose for the duration of the experiment.

**Table 1:** Summary statistics for cages of *An. stephensi *mosquitoes fed different estimated *P. berghei *ookinete densities

Experiment (date)	Estimated ookinete density fed (per μl)	Mean^‡ ^number of oocysts per mosquito on day 10* (range)	Mean^‡ ^sporozoite score^† ^per mosquito on day 22*	Total number of mosquitoes in each cage	Median survival (days) (95% C.I.)
**1**	0			442	32 (30, 34)
(August 2007)	100	46.4 (0-251)	2.4	399	ND (25, ND)
	400	141.8 (0-307)	3.0	645	26 (23, 31)
	2,000	259.6 (0-591)	3.7	562	21 (19, 24)
					
**2**	0			733	33 (32, 35)
(April 2008)	100	55.3 (0-212)	2.0	565	ND (ND, ND)
	400	83.1 (0-259)	2.6	662	34 (32, ND)
	2,000	138.4 (0-477)	1.8	502	30 (29, ND)
					
**3**	0			625	42 (41, ND)
(July 2008)	50	11.3 (0-47)	1.2	815	36 (35, 37)
	250	67.6 (0-121)	1.7	852	36 (34, 38)
	1,000	96.8 (0-200)	2.9	999	34 (32, 35)

^‡ ^Arithmetic mean parasite load in 20, randomly chosen surviving mosquitoes.

* Parasite densities presented from the dissections on days 10 and 22 as these are the standard days used in determining oocyst and salivary gland sporozoite density and therefore allow comparisons to be made with published studies, such as Sinden *et al *[47].

^† ^Sporozoites counted using a scoring system; 0, 0 sporozoites; 1, 1 to 10 sporozoites; 2, 11 to 100 sporozoites; 3, 101 to 1,000 sporozoites; 4, 1,001 to 10,000 sporozoites; 5, over 10,000 sporozoites.

ND, not determined as survival curve does not cross 0.5 and therefore the median survival cannot be calculated.

The populations of mosquitoes were followed over time post-feeding (which is also a proxy for mosquito age in this experiment) by recording the number of dead females in each group twice each day. In addition, for the first six days, and on alternate days after this time, a sample of twenty live mosquitoes from each group (with the exception of the control) were dissected to remove both the midgut and (from approximately day 10 onwards) salivary glands to determine parasite density in both midgut and salivary glands using fluorescence microscopy. This paper reports the results of the survival analysis. A summary of the resulting dynamics of parasite stages and densities with time post-feeding has been presented elsewhere [50].

### Statistical analysis

#### Non-parametric methods

Survival functions for each of the four groups (based on the ookinete density fed to the mosquitoes) in each of the three experiments, were estimated using the Kaplan-Meier estimate [51], classifying those mosquitoes lost to follow-up, e.g. those which were killed for dissection, as censored observations. (For details on the calculation of the Kaplan-Meier estimate see additional file 1: 'Detailed statistical methods'.)

The median survival time (with 95% confidence intervals) was calculated for each group to compare survival times, by determining the time beyond which 50% of the individuals in the population are expected to survive. The Mantel-Cox test and a log-rank test for trend were used to compare the survival distributions of the four groups within each of the experiments. The Mantel-Cox test is used for two-sample comparisons and is based on a test statistic with a chi-squared distribution and one degree of freedom under the null hypothesis that there is no difference between the survivorship of the individuals in the two groups under comparison [52,53] (for further details see additional file 1: 'Detailed statistical methods'). The log-rank test for trend was computed because the four groups to be compared in each experiment represented ordered, increasing, densities of infection. Therefore, the codes assigned to each of the mosquito groups were the number of ookinetes per μl of blood fed, which allowed this test to investigate if a linear trend exists between parasite density and survival. The resulting test statistic has a chi-squared distribution with one degree of freedom, under the null hypothesis of no trend across the groups [52,54] (for further details see additional file 1: 'Detailed statistical methods').

The combined datasets from the three experiments were analysed using Cox regression survival analysis (proportional hazards model) fitting ookinete density fed to the mosquitoes first as a categorical variable to test for differences in survival between cages, and subsequently as a continuous variable to explore the impact of an increase in parasite density on mosquito survival. This statistical analysis allows the impact of parasite density on mosquito survival to be tested whilst controlling for variation due to experiment.

#### Estimation of mosquito mortality rates

The modelling of survival data centers on the hazard function (the instantaneous death rate), which is used to express the risk or hazard of death at time *t*. Kaplan-Meier estimates, which assume that this hazard function is constant between successive death times, were calculated and plotted for the mid-point of each time-interval (for details as to their calculation see additional file 1: 'Detailed statistical methods').

Some of the most common hazard functions applied in survival analysis were used to explore the underlying mosquito survivorship. These included a constant death rate, the Gompertz function (the rate of mortality increases with age in such a manner that its logarithm is linearly proportional to age), and the Weibull function (the rate of mortality increases or decreases monotonically with age depending on the values of a shape and a scale parameter). However, as the observed hazard rates initially declined before increasing as time post-engorgement progressed, none of these functions were able to describe adequately the pattern observed in the data. Consequently, the following empirical quadratic hazard function for the relationship between mortality rate and time post-engorgement [55], was fitted by least squares estimation,(1)

This function describes a parabola, with parameter *θ *representing the mortality rate at the time of feeding (i.e. when *t *= 0), and parameters *δ *and *ν *being associated, respectively, with the subsequent decline and increase in death rate with time post-feeding, which could represent different biological causes of mortality. Parameters *ν *, *δ *and *θ *were each allowed to vary linearly with the density of ookinetes fed to the mosquitoes (*K*) to identify whether vector mortality is a density-dependent process. The full equation is therefore given as,(2)

where *ν*_0_, *δ*_0 _and *θ*_0 _represent the baseline hazard experienced by uninfected mosquitoes, and *ν*_1_, *δ*_1 _and *θ*_1 _represent the additional mortality per unit increase in ookinete density. Equation (2) was fitted to the full dataset using non-linear least squares estimation, and allowing the average mosquito mortality rate to vary between experiments to account for inter-experimental variability. Analysis of variance tests were conducted on nested versions of this full model to find the most parsimonious hazard function using the 'nls' and 'anova' commands in the statistical package R [56] as described by Bolker [57]. Ninety-five percent confidence intervals (95% C.I.) for the best-fit model were estimated using bootstrapping methods (see additional file 2: 'Generation of 95% confidence intervals for the best-fit model'). The survivorship function contains the integrated hazard function as detailed in additional file 1: 'Detailed statistical methods'.

#### Life expectancy

The median survival times (Table 1) provide an indication of life expectancy immediately after engorgement for each of the mosquito groups. In addition, life expectancy can be calculated using the parametric survivorship model described above, including each parameter of Equation (1) as a linear function of fed ookinete density as in equation (2):(3)

Life expectancy at *t *= 0 of a group of mosquitoes fed *K *ookinetes, *e*_0_(*K*), is this survival function integrated from the time of feeding to the maximum time post-engorgement lived by an engorged mosquito,(4)

Equation (4) was evaluated using the Berkeley Madonna numerical integration package (Version 8.0.1) [58] to calculate how the life expectancy of mosquitoes varied with the number of fed ookinetes and time post-engorgement. In the literature, mosquito life-span has been previously discussed in relation to oocyst rather than ookinete density, and therefore by way of illustration, mosquito life expectancy was also related to the mean oocyst load found in the sample of mosquitoes dissected from each of the cages 10 days post-bloodfeed as described in additional file 3: 'Calculating how life expectancy of mosquitoes varies with mean oocyst density on day 10 and time post-engorgement'.

## Results

Table 1 summarizes the data from each of the three experiments (approximately 400-1,000 female mosquitoes fed in each of the cages). The median survival time experienced by each of the cages of mosquitoes within each experiment shows a general trend towards a decrease in survival with an increase in average parasite load.

**Figure 2:**
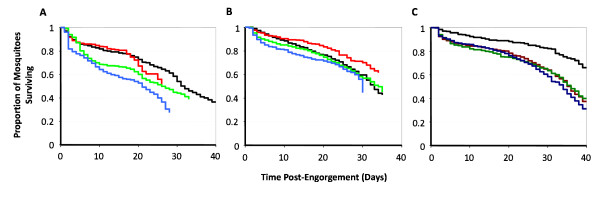
**Kaplan-Meier survival curves with time post-engorgement for each group of *An. stephensi *mosquitoes**. **(A) **Experiment 1. Colors; black = 0 ookinetes per μl of blood fed; red = 100 ookinetes per μl of blood fed; green = 400 ookinetes per μl of blood fed; blue = 2,000 ookinetes per μl of blood fed. **(B) **Experiment 2. Colors as in panel A: **(C) **Experiment 3. Colors; black = 0 ookinetes per μl of blood fed; dark red = 50 ookinetes per μl of blood fed; dark green = 250 ookinetes per μl of blood fed, dark blue = 1,000 ookinetes per μl of blood fed.

**Figure 3:**
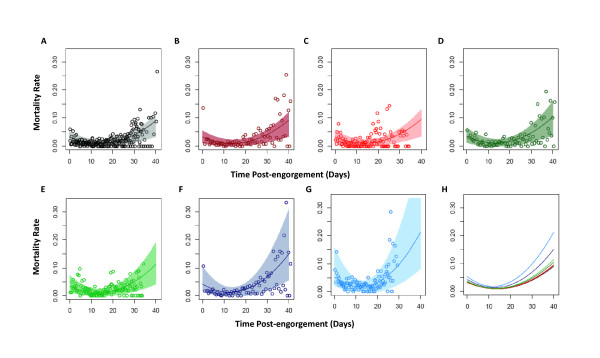
**Mortality rate with time post-engorgement**. Relationship between the mortality rate of *An. stephensi *mosquitoes fed on blood containing different densities of *P. berghei *ookinetes and time post-engorgement (days). Markers correspond to the observed death rates plotted for the mid-point of each time interval. The lines are the best fit hazard model defined in Equation (2) for each parasite density, and the shaded area corresponds to 95% confidence intervals. Panels A to G represent increasing ookinete density per μl of blood fed, with colors as in Figure 2; **(A) **Control (0 ookinetes). **(B) **50 ookinetes. **(C) **100 ookinetes. **(D) **250 ookinetes. **(E) **400 ookinetes. **(F) **1,000 ookinetes. **(G) **2,000 ookinetes. **(H) **Hazard curves from each of the parasite densities on a single axis to facilitate comparison; colors as in panels A to G, in order from lowest to highest at time post engorgement = zero (where curves cross the y-axis), 0, 50, 100, 250, 400, 1000 and 2000 ookinetes per μl of blood fed. Figure 4 illustrates how the parameters of the mortality function vary with ookinete density fed to the mosquitoes.

Figure 2 presents the observed proportion of mosquitoes surviving each time interval as Kaplan-Meier survival curves for every mosquito group in each of the three experiments. Mosquitoes in the control group of Experiment 1 were followed up until day 53 post-feeding, when every mosquito had died; the figures only display results up until day 40 to facilitate comparison with the other mosquito groups. The mosquitoes clearly experienced different mortality through the course of the experiment depending on which infection intensity group they belonged to (especially evident in experiments 1 and 3). In addition, if mosquito mortality were independent of age (here measured as time since feeding), these survival curves would be represented by an exponential decline. However, in each of the cages, including the control, the survival curves do not conform to an exponential distribution of survival times (Figure 2), and observed mortality rates are not constant with age (Figure 3), indicating that mosquitoes do senesce.

The log-rank test applied to each of the experiments had a significant chi-square value in all three cases (Table 2). The results of a series of Mantel-Cox tests, conducted as pair-wise comparisons between the survival of mosquitoes in each cage within each experiment, are given in Table 2. Experiment 1 indicates that the survival of mosquitoes in the low infection density group (100 ookinetes per μl of blood fed) did not differ significantly from that experienced by the control group, whereas mosquitoes in the intermediate (400 ookinetes per μl) and high density groups (2,000 ookinetes per μl) experienced statistically significantly (see Table 2) higher mortality in a dose-dependent manner (see Table 1 for mean numbers of oocysts and sporozoites in each population). The results from experiment 2 indicate that the mosquitoes in the low density group actually experienced a lower mortality rate than the control group, the intermediate density group did not differ significantly from the control group, and the highest parasite density group had significantly higher mortality than each of the other groups. The Mantel-Cox tests that compared the cages from experiment 3 indicate that all three groups fed on infectious blood experienced significantly more mortality than the control group fed on uninfected blood, but that there was no difference between the three infected groups. The Cox regression results summarized in Table 3, show that when combining the data from all three experiments, all ookinete densities fed to mosquitoes, apart from 100 ookinetes per μl, resulted in significantly greater mosquito mortality than the control blood containing no ookinetes. In addition, treating ookinete density as a continuous variable resulted in a significant p-value, suggesting an increase in mosquito mortality with ookinete density. The Cox regression analysis also indicated a significant effect of experiment on mosquito mortality, with mosquitoes in experiments 2 and 3 experiencing significantly lower levels of mortality than those in experiment 1.

**Table 2:** Results of two-sample and multi-sample statistical comparisons of *An. stephensi *survival times.

Experiment (Date)	Mosquito Group (Ookinete density per μl of blood)	Test Statistic	Statistical Results			
**1**			100	400	2,000	
				
(August 2007)	0	Hazard Ratio (HR)	1.1413	1.4261	2.2004	
		HR C.I.	(0.87, 1.50)	(1.17, 1.74)	(1.77, 2.74)	
		Mantel-Cox	0.9124	12.4278	52.6238	
		P-value	0.3395	0.0004*	<0.0001*	
						
	100	HR		1.4003	1.9702	
		HR C.I.		(1.09, 1.80)	(1.52, 2.55)	
		Mantel-Cox		6.9297	27.2606	
		P-value		0.0085*	<0.0001*	
						
	400	HR			1.4023	
		HR C.I.			(1.16, 1.70)	
		Mantel-Cox			12.2886	
		P-value			0.0005*	

	All 4 Groups	Log-rank				55.89
		P-value				<0.0001*

**2**			100	400	2,000	
				
(April 2008)	0	HR	0.7312	1.1786	1.7447	
		HR C.I.	(0.58, 0.92)	(0.97, 1.44)	(1.38, 2.20)	
		Mantel-Cox	7.0017	2.6747	22.3554	
		P-value	0.0081*	0.1020	<0.0001*	
						
	100	HR		1.6063	2.1607	
		HR C.I.		(1.27, 2.03)	(1.65, 2.82)	
		Mantel-Cox		15.7261	33.6528	
		P-value		<0.0001*	<0.0001*	
						
	400	HR			1.3186	
		HR C.I.			(1.05, 1.66)	
		Mantel-Cox			5.6392	
		P-value			0.0176*	
						
	All 4 Groups	Log-rank				31.20
		P-value				<0.0001*

**3**			50	250	1,000	
				
(July 2008)	0	HR	2.2198	2.1914	2.5451	
		HR C.I.	(1.77, 2.79)	(1.76, 2.73)	(2.06, 3.15)	
		Mantel-Cox	49.5610	51.1026	78.9376	
		P-value	<0.0001*	<0.0001*	<0.0001*	
						
	50	HR		1.0144	1.1677	
		HR C.I.		(0.86, 1.20)	(0.99, 1.37)	
		Mantel-Cox		0.0283	3.7481	
		P-value		0.8664	0.0529	
						
	250	HR			1.1543	
		HR C.I.			(0.99, 1.34)	
		Mantel-Cox			3.5264	
		P-value			0.0604	
						
	All 4 Groups	Log-rank				36.61
		P-value				<0.0001*

* Significant at 5% level

C.I., 95% confidence interval.

**Table 3:** Cox regression analysis results.

Variable	Coefficient	Standard Error	Hazard Ratio (95% C.I.)	P-value
**Ookinete density per μl of blood fed as a categorical variable**				
Control (0)	0	-	1	-
50	0.754	0.112	2.125 (1.707, 2.645)	<0.001*
100	-0.074	0.086	0.928 (0.784, 1.099	0.390
250	0.767	0.110	2.153 (1.737, 2.669)	<0.001*
400	0.314	0.067	1.369 (1.200, 1.561)	<0.001*
1000	0.887	0.106	2.429 (1.974, 2.988)	<0.001*
2000	0.639	0.071	1.894 (1.649, 2.176)	<0.001*

Experiment 1	0	-	1	-
Experiment 2	-0.411	0.053	0.663 (0.598, 0.735)	<0.001*
Experiment 3	-1.000	0.105	0.368 (0.299, 0.452)	<0.001*

**Ookinete density per μl of blood fed as a continuous variable**				
Control (0)	0	-	1	-
Ookinete density	3.26 × 10^-4^	2.88 × 10^-5^	1.00032 (1.0003, 1.0004)	<0.001*

Experiment 1	0	-	1	-
Experiment 2	-0.421	0.052	0.656 (0.593, 0.727)	<0.001*
Experiment 3	-0.474	0.046	0.622 (0.569, 0.680)	<0.001*

* Significant at 5% level

**Figure 4:**
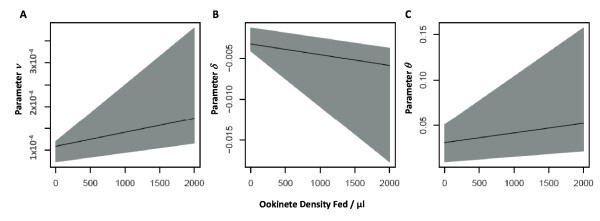
**Relationship between parameters of the mortality function and ookinete density fed**. Linear functions (as illustrated in Equation (2)) are fitted to the relationship between the parameter values of the mortality function and parasite density fed to each group of mosquitoes (ookinetes per μl of blood). Shaded areas represent 95% confidence intervals. **(A) **Parameter *ν*, which predominantly represents the increase in mortality rate with time-post feeding; parameter values (and 95% confidence intervals), *ν*_0 _= 1.18 × 10^-4 ^(4.65 × 10^-5^, 1.40 × 10^-4^)**, *ν*_1 _= 6.43 × 10^-8 ^(4.29 × 10^-8^, 2.60 × 10^-7^)**. **(B) **Parameter *δ*, which predominantly represents the initial decline in mortality rate with time post-feeding; *δ*_1 _= -3.27 × 10^-3 ^(-4.13 × 10^-3^, -1.31 × 10^-3^)**, *δ*_1 _= -1.30 × 10^-6 ^(-6.77 × 10^-6^, -1.24 × 10^-6^)*. **(C) **Parameter *θ*, which represents the mortality rate at the time of feeding; *θ*_0 _= 3.09 × 10^-2 ^(9.69 × 10^-3^, 5.06 × 10^-2^)**, *θ*_1 _= 1.07 × 10^-5 ^(5.99 × 10^-6^, 5.33 × 10^-5^)*. Significant p-values (* represents a p-value < 0.05 and ** represents a p-value < 0.001) indicate that the best-fit mortality function includes each of the parameter values in Equation (2).

Life tables for each mosquito group in each experiment are presented in additional file 4: 'Life tables for each experiment'. Mortality rates per time interval, and fitted by the parametric hazard function described in Equation (1), are plotted in Figures 3A to 3G (for the range of ookinete densities explored), and compared in Figure 3H. In general, mosquitoes experienced a degree of excess mortality immediately after feeding, and their death rate declined with time post-engorgement (age) to a minimum value before subsequently rising again, generating a parabolic shape. The empirical mortality function described in Equation (1) fitted this pattern well, the largest discrepancies occurring at the end of the experiments when few mosquitoes remained alive in each of the cages and therefore their survival or death resulted in larger fluctuations. The relationship between parameters *ν*, *δ *and *θ *of the mortality functions and the ookinete density fed to the mosquitoes (as in Equation (2)) is shown in Figures 4A, 4B and 4C respectively, and the results of statistical tests (on nested versions of the model, see Methods) indicate that each of these three parameters are significantly parasite-density dependent, with the inclusion of each of the parameters in Equation (2) significantly improving the fit of the model.

Allowing mortality rates to vary between the different experiments significantly improved the fit of the model to the observed data; mortality rates in the second and third experiments were on average 30% (95% C.I., 17-42%) and 32% (21-42%) lower than in experiment 1 respectively, suggesting substantial between-experiment variability.

**Figure 5:**
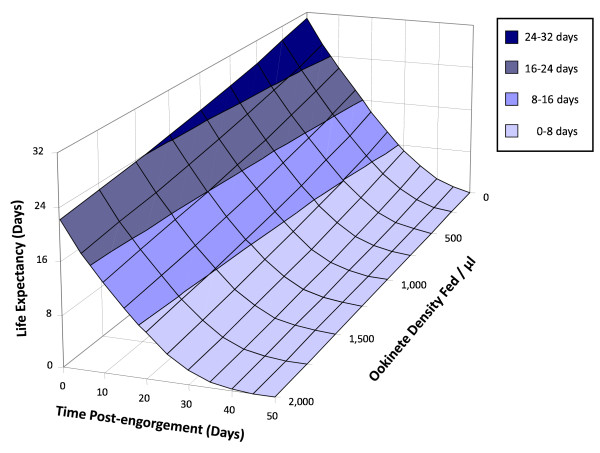
**Mosquito life expectancy**. Life expectancy of mosquitoes maintained in the laboratory, plotted against time post-engorgement and number of ookinetes per μl of blood fed to the mosquitoes. The life expectancy values are generated from Equation (4) with *S*(*t*, *K*) as defined in Equation (3).

Figure 5 shows a 3-dimensional plot of mosquito life expectancy (denoted *e*) as it varies with both time post-feeding (*t*) and ookinete density fed (*K*), i.e., *e*(*t*, *K*), using the mortality parameters estimated from combining the data from all three experiments as in Figure 4. Additional file 5: 'Mosquito life expectancy with time post-engorgement and mean number of oocysts on day 10 post-engorgement' relates this life expectancy to oocyst density 10 days post-engorgement as discussed in the Methods. This illustrates that life expectancy decreases with both parasite density and time post-engorgement.

## Discussion

### The role of model systems

Any single model system cannot accurately reflect the biology of all natural parasite-vector combinations. Nonetheless studies on the biology of *Plasmodium *spp per se, and their interactions with *Anopheles *mosquitoes have been advanced considerably by the analysis of *P. berghei *in *An. stephensi *[47]. This paper exploits the unique ability to study the effect of increasing densities of homogeneous populations of *P. berghei *(clone) on the survival of *An. stephensi *(inbred iso-female line) in a controlled biological environment. The results reported here indicate that, in captivity, *An. stephensi *mosquitoes experience initial blood feed-associated and age-dependent mortalities, and that their survival decreases with the intensity of *P. berghei *infection.

### Blood feeding- and age-dependent mortality

The time (age)-dependent curves of survivorship (Figure 2) and mortality rates (Figure 3) indicate that female mosquitoes have the potential to senesce, in agreement with previously published studies in a variety of species [10,11,16,17,59]. Most previous analyses have used the Gompertz hazard function. However, this was not adequate to describe the mortality rates experienced by the mosquitoes in the experiments presented here; in each of the mosquito groups, including the control, mortality rates were found to be high immediately after feeding, decreasing initially to a minimum before increasing with age. This functional form, depicted in Figure 3, which describes the mortality rates experienced by the mosquitoes, could result from a number of biological processes. The initial mortality (measured by parameter *θ *of the hazard function in Equation (1) of the Methods section) is likely to be in part associated with the act of feeding itself, for example, allowing the bacterial population within the mosquito midgut to proliferate [60]. Additionally, this early mortality in the control group might also be attributed to asexual stages of the parasite, or parasite-induced factors present in the mouse blood up-regulating the mosquito's immune system [61], which could be costly to the survival of the mosquito. After reaching a minimum mortality rate at an intermediate time post-engorgement, the increase in mortality rate (measured primarily by parameter *ν *of the hazard function in Equation (1)) is expected to represent the effect of mosquito ageing.

These results suggest that the age at which a mosquito bites an infectious host is important in determining the probability that it will transmit the parasite and contribute to malaria transmission. Mosquitoes exhibiting age-dependent mortality patterns are more likely to transmit pathogens if they bite an infectious host when their mortality rate is at a minimum, as they are more likely to survive the extrinsic incubation period. Clements and Patterson [17] and Styer *et al *[16] illustrated the importance of accepting this concept of mosquito senescence, showing that the longevity factor [3] and the vectorial capacity for a variety of mosquito species can be significantly overestimated if calculated using the simpler exponential hazard model compared to a hazard model which is age-dependent such as the Gompertz model. Consequently, the potential impact of anti-vectorial control measures could be underestimated by assuming age-independent mosquito mortality. Gillies [62] even called for the exponential hazard model that assumes no senescence to be 'buried', as it produces results which are 'at best approximations'. Recent studies that explore the potential impact of novel control strategies such as fungal biopesticide sprays have acknowledged this by using a mosquito age-structured model and adult female age (time since infection)-dependent mortality [63].

The study of mosquito cohorts in the laboratory presented here provides patterns of mortality and survival under conditions in which many individuals may survive until old age, and therefore represent the baseline state which is inevitably modified on exposure to natural conditions. It is particularly important to determine whether age-dependent mortality is relevant in field situations as it has previously been accepted that few organisms die of senescence in nature, with the majority being killed by other hazards such as predators or disease before they reach 'old age' [64]. Previous research has found both constant survival rates in natural settings in *An. gambiae *(using Polovodova age-grading) [9,41], and increasing death rates with insect age in many mosquito species in the field [12,17,59]. A conceptual shift from age-independent to age-dependent mortality rates and an understanding of their relative merits in natural malaria transmission settings requires detailed knowledge of mosquito population age structure and its relation to pathogen transmission dynamics. Current age-grading techniques used in the field are most commonly based on morphological changes in the mosquito, such as the detection of tracheal skeins, which only permits differentiation between nulliparous and parous females [65], or the enumeration of follicular relics for the assessment of physiological age [66], which is difficult to implement in the field (requiring training and the use of phase-contrast microscopy), as discussed by Hugo *et al*, 2008 [67]. There is therefore a need for the development of novel age-grading assays that allow investigation of the age structure in mosquito populations prior to and after interventions. Additionally, other age-related changes also occur in mosquitoes, such as changes in flight performance [68], structure of the salivary glands [69], immune function [70,71], and efficiency of detoxification mechanisms [72,73], showing that mosquitoes, like other organisms, experience age-related structural and functional deterioration.

### *Plasmodium*-dependent mortality

Ferguson and Read's review [38] illustrated the inconsistent results from research aiming to elucidate the impact of *Plasmodium *infection on mosquito mortality. This review also indicated a lack of systematic research to understand the effect of increasing parasite density on mosquito survival, despite it often being postulated that *Plasmodium *is only harmful to the vector when parasite loads are very high [28,35]. The results presented here indicate that mosquito mortality was influenced by the range of intensities of *Plasmodium *infection explored. In general, the higher the parasite density fed to the mosquitoes the greater the mortality experienced as indicated by the Cox regression results (Table 3). In the first two experiments, mosquito survival in the group with the lowest *Plasmodium *density (100 ookinetes/μl of blood; see Table 1 for resulting oocyst and sporozoite densities) was found either not to differ from, or to be lower than, that in the control group, whereas significant differences were found with higher parasite densities, in a dose-dependent manner. This appears to be consistent with the few previous studies that have considered infection density [33,37,46,74]. In particular, Klein *et al *[37] found that the survival rates of *Anopheles dirus *with less than 10 *Plasmodium cynomolgi *oocysts were not significantly different from those in uninfected mosquitoes, whereas the mean survival rates of the groups infected with over 41 oocysts per mosquito were significantly lower. Interestingly, the results from the third experiment in this paper indicated that even very low parasite densities (50 ookinetes per μl of blood fed, which (from Table 1) resulted in a mean oocyst load of 11 per mosquito on day 10) can reduce mosquito survival. The fitness cost to mosquitoes of feeding on infected versus uninfected hosts may even be higher than estimated in this experimental design, as the control group was fed on blood with asexual parasitaemia, which by potentially eliciting costly immune responses, could result in increased mosquito mortality [61].

The impact of parasite density on mosquito mortality is further exemplified by the relationship between the parameter values of the empirical hazard function describing how mortality rates change over time (Equation (2) in the Methods section) and parasite density fed to each of the mosquito groups (Figure 4). Parameter *θ *(the intercept), which describes the rate of mortality immediately post-feeding, varies with parasite density, being lowest in uninfected mosquitoes and increasing with parasite density fed to the mosquito. This indicates that this initial mortality may not only be associated with bacteria proliferating in the midgut as discussed above, but that there is also an impact of, or interaction with, *Plasmodium *infection, potentially due to rupture of the midgut causing septic injury, particularly at high parasite densities when the ability of the midgut to repair and seal [75,76] may be compromised (as seen in Figure 3 of [77]). Parameter *δ*, which primarily measures the degree of the subsequent decline in the mortality rate, decreases with parasite density, indicating a steeper decrease in mortality with increasing parasite density (due to starting from a higher intercept). Finally, parameter *ν*, which measures predominantly the slope of the final rise in mortality rate with time post-feeding, is positively and significantly associated with infection density, indicating that mortality not only increases with age, but that the rate of this increase is amplified by the intensity of *Plasmodium *infection. This suggests that *Plasmodium *density has the potential of affecting the shape of the hazard function of infected *Anopheles *mosquitoes over their full life-span. Interestingly, no specific change in mortality rate was seen on day 12 or 14 when sporozoites were first found in the salivary glands of the sample of mosquitoes dissected from each of the groups [50].

These results reveal that under laboratory conditions, *Anopheles *mortality is not only influenced by *Plasmodium *infection, but that this may also be an important source of density dependence in the system. This may, therefore, go someway towards explaining the varied and often conflicting results found in the past and reviewed by Ferguson and Read [38]. The majority of previous experiments had not explored or even reported parasite density, and therefore the density used in each study may explain why some have found evidence for *Plasmodium*-induced mosquito mortality whilst others have not. Additionally, *Plasmodium*-density dependent mortality has the potential to explain the low oocyst loads found in the field as those mosquitoes with large numbers of oocysts may have died as a result of infection as well as, or because of an interaction with, environmental factors. Density-dependent, parasite-induced vector mortality has been reported in other vector-borne diseases, and particularly in the filarial parasites, both in captivity [55,78,79] and in the field [80,81].

It is recognized that there are a number of possible mechanisms and stages during *Plasmodium *development in which infection could damage the vector and therefore increase mortality. There is mixed evidence for many of these potential mechanisms, as discussed below, and it is possible that all could be exacerbated or altered in some way by the density of infection. The parasite can cause physical tissue damage, for example ookinetes perforating the mosquito midgut, and this could also increase susceptibility to bacterial infection and/or invasion by other parasites [82,83]. However, the 'time bomb' theory suggests that as the parasite passes through the midgut wall it initiates apoptosis and expulsion of the midgut cell, which is accompanied by a sealing of the midgut epithelium which regains integrity and a healthy appearance within 48 hours [75,76]. As mentioned above, this seal may not be entirely aseptic, allowing bacterial infection to spread, especially during an infection with high parasite numbers when the ability of the midgut to repair may be compromised or slowed. It has also been postulated that *Plasmodium *infection may lead to resource depletion in the mosquito as levels of amino acids in their haemolymph have been shown to be reduced, and glucose usage has shown to be up to eight times as much as in uninfected mosquitoes [84,85]. In contrast however, Rivero and Ferguson (2003) [86] found no evidence of a parasite-associated reduction in the energetic budget of mosquitoes. Additionally, since infection may be associated with a reduction in egg production [34,35], which is expensive in terms of resources, infection may even result in a saving of nutrients. In addition, mosquitoes have been shown to mount a variety of immune responses to pathogens [87-89], which can be energetically costly, incurring reproductive costs [90-92]. Such costly immune responses may be induced by the *Plasmodium *infection itself and/or by the increase in gut bacteria due to blood feeding [93].

The feeding behaviour of infected mosquitoes has also been shown to differ between uninfected and infected mosquitoes, with infected mosquitoes spending more time feeding, probing more regularly, more likely taking multiple blood meals, being more persistent feeders, and having poorer flight ability [94-98]. These additional behavioural changes can increase the mortality of infected mosquitoes whilst feeding in the field [39], and can vary temporally with the developmental stage of the parasite, balancing opportunities for *Plasmodium *transmission with the risk of feeding-associated mortality [94]. Laboratory studies have reported that the feeding persistence of female *An. stephensi *is decreased in the presence of *Plasmodium yoelii nigeriensis *oocysts, but increased when the malaria has developed into transmissible sporozoites in the salivary glands [94]. Laboratory experiments, such as those reported here, exclude these possible indirect costs of infection such as increased risk of predation, and therefore the effect of infection on mosquito mortality may be more pronounced in the field compared to the laboratory due to greater levels of environmental stress. Insects in this study were fed only once and subsequently kept under controlled laboratory conditions, instead of undergoing their natural gonotrophic cycle of feeding, oviposition, and host-seeking, which may additionally impact on their chances of survival.

The work reported here was carried out using an experimental vector-parasite combination, the model system *P. berghei-An. stephensi*, which allowed the investigation to be conducted under tightly controlled conditions. The average oocyst numbers resulting from the ookinete densities fed to the mosquitoes in this study are higher than the average number of *Plasmodium falciparum *oocysts found in *Anopheles gambiae *in the field, and therefore the density dependence found may not be as evident in studies of vector-parasite combinations found naturally. Additionally, it is recognized that the analysis of vector-parasite combinations not naturally found in the field may increase the chance of finding evidence of *Plasmodium*-induced vector mortality, as stated by Ferguson and Read [38]. As well as this perhaps resulting from a lack of parasite-vector co-adaptation, it may also be due to the greater likelihood of distinguishing parasite-induced effects from environmental risks under the more controlled conditions of the laboratory. In this study the removal of extraneous variables has permitted the unequivocal identification of density-dependent *Plasmodium *induced *Anopheles *mortality, and therefore, as in Sinden *et al *[47], the results have generated testable hypotheses, which now should be followed up with studies of other *Plasmodium-Anopheles *combinations, including the less tractable human malaria parasites and their multiple vector species, both in the laboratory and in the field.

## Conclusions and implications for malaria transmission and control

These results indicate that, in the model system investigated, the life expectancy of *Anopheles *mosquitoes is dependent on both insect age and the density of *Plasmodium *infection, as depicted in Figure 5. This emphasizes the importance of testing these hypotheses in combinations of medical importance, and of understanding the impact of these factors on mosquito mortality, as they influence the probability of a mosquito surviving the extrinsic incubation period and contributing to malaria transmission. Linking these results to previous findings [47] (which illustrated density-dependent transitions between sporogony parasite stages), indicates that intermediate 'optimum' parasite densities may exist for the parasite to complete transmission, and it is likely that these optima will depend on the specific *Plasmodium-Anopheles *combination. Understanding such intricacies is of utmost importance, as it is possible that interventions could have unexpected outcomes; reducing high parasite load for example, could inadvertently increase the life expectancy of the vector and relax the density-dependent constraints operating upon sporogony within the vector, facilitating successful transmission of the pathogen. As a result it is important for studies of transmission-blocking strategies to report efficacy in terms of reductions in prevalence as well as parasite density to facilitate understanding of the impact of such interventions on malaria transmission.

As vector mortality is a particularly sensitive component of pathogen transmission, quantitative models seeking to describe transmission dynamics within the vector that do not include these processes could produce misleading results or miss epidemiologically important outcomes. The results presented here suggest that high parasite loads have the potential to reduce vector competence (summarized as the per capita probability of an ingested gametocyte to generate infectiousness) and vectorial capacity (which includes the daily probability of vector survival and the expectation of infective life or 'longevity factor'). Age- and parasite density-dependent mosquito mortality, as well as density-dependent *Plasmodium *development are in the process of being included into mathematical models that will provide a more comprehensive description of the processes that influence sporogony in the mosquito and the expectation of infective life. The usefulness of such models for the design, development, and evaluation of transmission-blocking strategies will be reported elsewhere.

## Competing interests

The authors declare that they have no competing interests.

## Authors' contributions

EJD, RES and M-GB conceived and designed the experiments. EJD and SZ performed the experiments. EJD and TSC analysed the data. EJD, TSC, RES and M-GB wrote the paper. All authors read and approved the manuscript.

## Supplementary Material

Additional file 1 **Detailed statistical methods**. Details of the calculation of the Kaplan-Meier survival function, Mantel-Cox test, log-rank test for trend, Kaplan-Meier hazard function and survival function. Click here for file

Additional file 2 **Generation of 95% confidence intervals for the best-ft model**. Details of how confidence intervals were calculated for the best-fit model using bootstrapping methods. Click here for file

Additional file 3 **Calculating how life-expectancy of mosquitoes varies with mean oocyst density on day 10 and time post-engorgement**. Methodology for alternative analyses, relating mosquito life-expectancy to oocyst rather than ookinete density; results are shown in Additional file 5. Click here for file

Additional file 4 **Life tables for each experiment**. Series of life tables describing the number of mosquitoes surviving, the number dead, and the corresponding mortality rate at each timepoint in each of the three experiments. Click here for file

Additional file 5 **Mosquito life expectancy with time post-engorgement and mean number of oocysts on day 10 post-engorgement**. Graph illustrating how mosquito life expectancy depends upon time post-engorgement and the mean number of oocysts per mosquito 10 days post bloodfeed. Click here for file
