# Supplementary material for: Anopheles mortality is both age- and Plasmodium-density dependent: implications for malaria transmission
Source: Malar J. 2009 Oct 12;8:228. doi: 10.1186/1475-2875-8-228 (PMC2770541; doi:10.1186/1475-2875-8-228)
Supplement: Additional file 1 — Detailed statistical methods. Details of the calculation of the Kaplan-Meier survival function, Mantel-Cox test, log-rank test for trend, Kaplan-Meier hazard function and survival function. [file 1475-2875-8-228-S1.DOC]

**Additional file 1: Detailed Statistical Methods**

***Calculating the Kaplan-Meier survival function****.* The times at which mosquitoes die are denoted by *t_j_*, for *j* = 1,2,...*r* (*r* representing the total number of ‘death times’), and the time intervals between these ‘death times’ are denoted *t_k_* to *t_k_*_+1_, for *k* = 1,2,...*r*. The Kaplan-Meier estimate (also known as the product-limit estimate) is therefore given by,

$$\mathop{S}\limits^{\wedge} (t)=\prod_{j=1}^{k} \left(\frac{n_{j}-d_{j}}{n_{j}}\right)$$

with *n_j_* equal to the number of mosquitoes which are alive, and therefore at risk of death, just before time *t_j_*, and *d_j_* equal to the number of deaths at time *t_j_*.

***The Mantel-Cox test*** is based on the test statistic,

$$W_{L}=U_{L}^{2}/V_{L}$$

which has a chi-squared distribution with one degree of freedom under the null hypothesis that there is no difference between the survivorship of the individuals in the two groups under comparison. The numerator is the statistic,

$$U_{L}=\sum_{j=1}^{r} (d_{1j}-e_{1j})$$

where *d*_1_*_j_* represents the number of deaths at time *t_j_* in the first group, and the expected number of deaths in group 1 is given by $e_{1j}=n_{1j}d_{j}/n_{j}$. The denominator is the variance of this statistic,

$V_{L}=\sum_{j=1}^{r} v_{1j}$,

where $v_{1j}=\frac{n_{1j}n_{2j}d_{j}(n_{j}-d_{j})}{n_{j}^{2}(n_{j}-1)}$, *n_j_* and *d_j_* are as above, and the subscripts 1 and 2 represent the two groups being compared [46, 47].

***The log-rank test for trend*** across *g* ordered groups is based on the test statistic,

$$W_{T}=U_{T}^{2}/V_{T}$$

which has a chi-squared distribution with one degree of freedom under the null hypothesis of no trend across the *g* groups. The numerator is the statistic

$$U_{T}=\sum_{i=1}^{g} w_{i}\left(d_{i.}-e_{i.}\right)$$

with $d_{i.}=\sum_{j=1}^{r_{i}} d_{ij}$ and $e_{i.}=\sum_{j=1}^{r_{i}} e_{ij}$ (*d_ij_* and *e_ij_* denoting respectively the observed and the expected number of deaths in the *i*th group (*i* = 1, …, *g*) at time *t_j_*). *r_i_* is the maximum time mosquitoes were alive until in the *i*th group, and *w_i_* represents a code assigned to the *i*th group. The codes assigned to each of the mosquito groups represented the number of ookinetes per μl in the blood on which they were fed, i.e. 0, 100, 400 and 2,000 for experiments 1 and 2 (and 0, 50, 250 and 1,000 in experiment 3), to allow the log-rank test for trend to test the effect of parasite density on survival. The denominator is the variance of *U_T_* which is given by,

where $\bar{w}$ is a sum of the quantities *w_i_*, weighted by the expected numbers of deaths; $\bar{w}=\frac{\sum_{i=1}^{g} w_{i}e_{i.}}{\sum_{i=1}^{g} e_{i.}}$.

***Calculating the Kaplan-Meier hazard function.*** The Kaplan-Meier estimate of the hazard function is given by,

$$\mu(t)=\frac{d_{j}}{n_{j}\tau_{j}}$$

where *d_j_* and *n_j_* are as above, and *τ_j_* = *t*(*_j_*_+1)_ – *t_j_*.

***Calculating the survival function from the mortality function.*** Integrating the mortality function in equation [1] yields *H*(*t*), the integrated hazard,

$H(t)=\int\mu(t) dt=\int(at^{2}+bt+c) dt$ [i]

of which survivorship is a function,

$\begin{matrix} \\ S(t)=\exp\left[-H(t)\right] \\ \end{matrix}$ [ii]

Combining Equations [i] and [ii] gives:

$S(t)=\exp\left[-\left(\frac{at^{3}}{3}+\frac{bt^{2}}{2}+ct\right)\right]$. [iii]
