# Supplementary material for: Anopheles mortality is both age- and Plasmodium-density dependent: implications for malaria transmission
Source: Malar J. 2009 Oct 12;8:228. doi: 10.1186/1475-2875-8-228 (PMC2770541; doi:10.1186/1475-2875-8-228)
Supplement: Additional file 2 — Generation of 95% confidence intervals for the best-ft model. Details of how confidence intervals were calculated for the best-fit model using bootstrapping methods. [file 1475-2875-8-228-S2.DOC]

**Additional file 2: Generation of 95% confidence intervals for the best-fit model**

Conservative 95% confidence interval estimates for the best-fit model were generated using bootstrapping methods by resampling (with replacement) within and between cages of the observed dataset (i.e. daily mortality rates were randomly selected from within each cage of mosquitoes, and 12 cages were randomly selected from the different experiments conducted and parasite densities explored). Equation [2] was then fitted to the resampled dataset using least squares estimation. This process was repeated 100,000 times. Lower and upper confidence interval curves were found which bound 95% of all of the best fit curves at each time point.
