# Supplementary material for: Anopheles mortality is both age- and Plasmodium-density dependent: implications for malaria transmission
Source: Malar J. 2009 Oct 12;8:228. doi: 10.1186/1475-2875-8-228 (PMC2770541; doi:10.1186/1475-2875-8-228)
Supplement: Additional file 3 — Calculating how life-expectancy of mosquitoes varies with mean oocyst density on day 10 and time post-engorgement. Methodology for alternative analyses, relating mosquito life-expectancy to oocyst rather than ookinete density; results are shown in Additional file 5. [file 1475-2875-8-228-S3.DOC]

**Additional file 3: Calculating how life-expectancy of mosquitoes varies with mean oocyst density on day 10 and time post-engorgement**

Parameters *ν*, *δ*, and *θ* of the mortality function defined in Equation [1] were each allowed to vary linearly with the mean density of oocysts 10 days post-engorgement (*C*). The full equation is therefore given as,

$\mu\left(t,C\right)=\left[\left(\nu_{0}+\nu_{1}C\right)t^{2}+\left(\delta_{0}+\delta_{1}C\right)t+\left(\theta_{0}+\theta_{1}C\right)\right]$ [A]

where *ν* _0_, *δ* _0_ and *θ* _0_ represent the baseline hazard experienced by uninfected mosquitoes, and *ν*_1_, *δ*_1_ and *θ*_1_ represent the additional mortality per unit increase in oocyst density. Equation [A] was fit to the full dataset using least squares estimation, allowing the average vector mortality rate to vary between experiments to account for inter-experimental variability. Integrating equation [A] allows the survivorship function to be obtained,

$S(t,K)=\exp\left\{-\left[(\nu_{0}+\nu_{1}C)\frac{t^{3}}{3}+(\delta_{0}+\delta_{1}C)\frac{t^{2}}{2}+(\theta_{0}+\theta_{1}C)t\right]\right\}$. [B]

Life expectancy at *t* = 0 of a group of mosquitoes with a mean *C* oocysts on day 10 post-engorgement, *e*_0_(*C*), is this survival function integrated from the time of feeding to the maximum time post-engorgement lived by an engorged mosquito,

$e_{0}(C)=\int_{0}^{\max} S(t,C)dt$. [C]

Equation [C] was evaluated using the Berkeley Madonna numerical integration package [52] to calculate how the life expectancy of mosquitoes varies with the mean number of oocysts 10 days post feeding and time post-engorgement, and the results are given in additional file 5: ‘Mosquito life expectancy with time post-engorgement and mean number of oocysts on day 10 post-engorgement’.
