# Supplementary material for: Anopheles mortality is both age- and Plasmodium-density dependent: implications for malaria transmission
Source: Malar J. 2009 Oct 12;8:228. doi: 10.1186/1475-2875-8-228 (PMC2770541; doi:10.1186/1475-2875-8-228)
Supplement: Additional file 4 — Life tables for each experiment. Series of life tables describing the number of mosquitoes surviving, the number dead, and the corresponding mortality rate at each timepoint in each of the three experiments. [file 1475-2875-8-228-S4.DOC]

**Additional file 4 – Life tables for each experiment**

Life table for *An. stephensi* mosquitoes fed on blood containing different *P. berghei* ookinete densities in experiment 1. *N*(*t*), number of mosquitoes alive at the beginning of timepoint; *S*(*t*), Kaplan Meier survival function; *μ*(*t*), mortality rate mosquito^-1^ day^-1^.

|  | | |  |  | | | | |  |  | | |  |  | | |  |  | | |
| --- | --- | --- | --- | --- | --- | --- | --- | --- | --- | --- | --- | --- | --- | --- | --- | --- | --- | --- | --- | --- |
| **Ookinete density per μl of blood fed to mosquito group** | | |  | **0** | | | | |  | **100** | | |  | **400** | | |  | **2,000** | | |
|  | | |  |  | | |  |  |  |  |  |  |  |  |  |  |  |  |  |  |
| **Time post engorgement and midpoint (*t* ) (Days)** | | |  | ***N*(*t* )** | | | ***S*(*t* )** | ***μ*(*t* )** |  | ***N*(*t* )** | ***S*(*t* )** | ***μ*(*t* )** |  | ***N*(*t* )** | ***S*(*t* )** | ***μ*(*t* )** |  | ***N*(*t* )** | ***S*(*t* )** | ***μ*(*t* )** |
|  | |  |  | |  | |  |  |  |  |  |  |  |  |  |  |  |  |  |  |
| 0 | 0.5 |  |  | | 442 | | 1 | 0.029 |  | 399 | 1 | 0.010 |  | 645 | 1 | 0.006 |  | 562 | 1 | 0.039 |
| 1 | 1.5 |  |  | | 429 | | 0.971 | 0.051 |  | 375 | 0.999 | 0.075 |  | 621 | 0.994 | 0.055 |  | 520 | 0.961 | 0.150 |
| 2 | 2.5 |  |  | | 407 | | 0.921 | 0.027 |  | 327 | 0.916 | 0.031 |  | 567 | 0.939 | 0.023 |  | 422 | 0.817 | 0.028 |
| 3 | 3.5 |  |  | | 396 | | 0.896 | 0.028 |  | 297 | 0.888 | 0.013 |  | 534 | 0.918 | 0.051 |  | 390 | 0.794 | 0.023 |
| 4 | 4.5 |  |  | | 385 | | 0.871 | 0.013 |  | 273 | 0.876 | 0.011 |  | 487 | 0.871 | 0.080 |  | 361 | 0.775 | 0.017 |
| 5 | 5.5 |  |  | | 380 | | 0.860 | 0.008 |  | 250 | 0.866 | 0.008 |  | 428 | 0.802 | 0.042 |  | 335 | 0.762 | 0.027 |
| 6 | 6.5 |  |  | | 377 | | 0.853 | 0.008 |  | 228 | 0.860 | 0.000 |  | 390 | 0.768 | 0.041 |  | 306 | 0.742 | 0.036 |
| 7 | 7.5 |  |  | | 374 | | 0.846 | 0.016 |  | 228 | 0.860 | 0.004 |  | 374 | 0.736 | 0.027 |  | 295 | 0.715 | 0.027 |
| 8 | 8.5 |  |  | | 368 | | 0.833 | 0.008 |  | 207 | 0.856 | 0.010 |  | 344 | 0.717 | 0.023 |  | 267 | 0.696 | 0.045 |
| 9 | 9.5 |  |  | | 365 | | 0.826 | 0.011 |  | 205 | 0.847 | 0.010 |  | 336 | 0.700 | 0.021 |  | 255 | 0.664 | 0.035 |
| 10 | 10.5 |  |  | | 361 | | 0.817 | 0.017 |  | 183 | 0.839 | 0.005 |  | 309 | 0.685 | 0.003 |  | 226 | 0.641 | 0.027 |
| 11 | 11.5 |  |  | | 355 | | 0.803 | 0.006 |  | 182 | 0.835 | 0.016 |  | 308 | 0.683 | 0.013 |  | 220 | 0.624 | 0.023 |
| 12 | 12.5 |  |  | | 353 | | 0.799 | 0.008 |  | 159 | 0.821 | 0.006 |  | 284 | 0.674 | 0.000 |  | 195 | 0.610 | 0.021 |
| 13 | 13.5 |  |  | | 350 | | 0.792 | 0.000 |  | 158 | 0.816 | 0.006 |  | 284 | 0.674 | 0.004 |  | 191 | 0.597 | 0.010 |
| 14 | 14.5 |  |  | | 350 | | 0.792 | 0.011 |  | 137 | 0.811 | 0.007 |  | 263 | 0.672 | 0.011 |  | 169 | 0.591 | 0.030 |
| 15 | 15.5 |  |  | | 346 | | 0.783 | 0.006 |  | 136 | 0.805 | 0.000 |  | 260 | 0.664 | 0.004 |  | 164 | 0.574 | 0.018 |
| 16 | 16.5 |  |  | | 344 | | 0.778 | 0.006 |  | 116 | 0.805 | 0.034 |  | 239 | 0.662 | 0.013 |  | 141 | 0.563 | 0.007 |
| 17 | 17.5 |  |  | | 342 | | 0.774 | 0.018 |  | 112 | 0.777 | 0.036 |  | 236 | 0.653 | 0.013 |  | 140 | 0.559 | 0.007 |
| 18 | 18.5 |  |  | | 336 | | 0.760 | 0.015 |  | 88 | 0.749 | 0.034 |  | 213 | 0.645 | 0.033 |  | 119 | 0.555 | 0.025 |
| 19 | 19.5 |  |  | | 331 | | 0.749 | 0.027 |  | 85 | 0.724 | 0.071 |  | 206 | 0.624 | 0.039 |  | 116 | 0.541 | 0.026 |
| 20 | 20.5 |  |  | | 322 | | 0.729 | 0.034 |  | 59 | 0.673 | 0.068 |  | 178 | 0.600 | 0.056 |  | 93 | 0.527 | 0.065 |
| 21 | 21.5 |  |  | | 311 | | 0.704 | 0.006 |  | 55 | 0.627 | 0.036 |  | 168 | 0.566 | 0.024 |  | 87 | 0.493 | 0.046 |
| 22 | 22.5 |  |  | | 309 | | 0.699 | 0.026 |  | 33 | 0.604 | 0.000 |  | 144 | 0.553 | 0.035 |  | 63 | 0.470 | 0.048 |
| 23 | 23.5 |  |  | | 301 | | 0.681 | 0.020 |  | 33 | 0.604 | 0.000 |  | 139 | 0.533 | 0.007 |  | 60 | 0.448 | 0.033 |
| 24 | 24.5 |  |  | | 295 | | 0.667 | 0.014 |  | 13 | 0.604 | 0.077 |  | 118 | 0.530 | 0.034 |  | 38 | 0.433 | 0.079 |
| 25 | 25.5 |  |  | | 291 | | 0.658 | 0.021 |  | 12 | 0.558 |  |  | 114 | 0.512 | 0.026 |  | 35 | 0.399 | 0.086 |
| 26 | 26.5 |  |  | | | 285 | 0.645 | 0.021 |  |  |  |  |  | 91 | 0.498 | 0.033 |  | 12 | 0.365 | 0.167 |
| 27 | 27.5 |  |  | | | 279 | 0.631 | 0.032 |  |  |  |  |  | 88 | 0.482 | 0.023 |  | 10 | 0.304 |  |
| 28 | 28.5 |  |  | | | 270 | 0.611 | 0.044 |  |  |  |  |  | 66 | 0.471 | 0.045 |  |  |  |  |
| 29 | 29.5 |  |  | | | 258 | 0.584 | 0.074 |  |  |  |  |  | 63 | 0.449 | 0.016 |  |  |  |  |
| 30 | 30.5 |  |  | | | 239 | 0.541 | 0.071 |  |  |  |  |  | 42 | 0.442 | 0.024 |  |  |  |  |
| 31 | 31.5 |  |  | | | 222 | 0.502 | 0.050 |  |  |  |  |  | 41 | 0.432 | 0.049 |  |  |  |  |
| 32 | 32.5 |  |  | | | 211 | 0.477 | 0.038 |  |  |  |  |  | 19 | 0.411 | 0.053 |  |  |  |  |
| 33 | 33.5 |  |  | | | 203 | 0.459 | 0.030 |  |  |  |  |  | 18 | 0.389 |  |  |  |  |  |
| 34 | 34.5 |  |  | | | 197 | 0.446 | 0.036 |  |  |  |  |  |  |  |  |  |  |  |  |
| 35 | 35.5 |  |  | | | 190 | 0.430 | 0.047 |  |  |  |  |  |  |  |  |  |  |  |  |
| 36 | 36.5 |  |  | | | 181 | 0.410 | 0.022 |  |  |  |  |  |  |  |  |  |  |  |  |
| 37 | 37.5 |  |  | | | 177 | 0.400 | 0.045 |  |  |  |  |  |  |  |  |  |  |  |  |
| 38 | 38.5 |  |  | | | 169 | 0.382 | 0.047 |  |  |  |  |  |  |  |  |  |  |  |  |
| 39 | 39.5 |  |  | | | 161 | 0.364 | 0.062 |  |  |  |  |  |  |  |  |  |  |  |  |
| 40 | 40.5 |  |  | | | 151 | 0.342 | 0.066 |  |  |  |  |  |  |  |  |  |  |  |  |
| 41 | 41.5 |  |  | | | 141 | 0.319 | 0.078 |  |  |  |  |  |  |  |  |  |  |  |  |
| 42 | 42.5 |  |  | | | 130 | 0.294 | 0.069 |  |  |  |  |  |  |  |  |  |  |  |  |
| 43 | 43.5 |  |  | | | 121 | 0.274 | 0.124 |  |  |  |  |  |  |  |  |  |  |  |  |
| 44 | 44.5 |  |  | | | 106 | 0.240 | 0.132 |  |  |  |  |  |  |  |  |  |  |  |  |
| 45 | 45.5 |  |  | | | 92 | 0.208 | 0.152 |  |  |  |  |  |  |  |  |  |  |  |  |
| 46 | 46.5 |  |  | | | 78 | 0.176 | 0.115 |  |  |  |  |  |  |  |  |  |  |  |  |
| 47 | 47.5 |  |  | | | 69 | 0.156 | 0.188 |  |  |  |  |  |  |  |  |  |  |  |  |
| 48 | 48.5 |  |  | | | 56 | 0.127 | 0.143 |  |  |  |  |  |  |  |  |  |  |  |  |
| 49 | 49.5 |  |  | | | 48 | 0.109 | 0.188 |  |  |  |  |  |  |  |  |  |  |  |  |
| 50 | 50.5 |  |  | | | 39 | 0.088 | 0.256 |  |  |  |  |  |  |  |  |  |  |  |  |
| 51 | 51.5 |  |  | | | 29 | 0.066 | 0.241 |  |  |  |  |  |  |  |  |  |  |  |  |
| 52 | 52.5 |  |  | | | 22 | 0.050 | 0.455 |  |  |  |  |  |  |  |  |  |  |  |  |
| 53 | 53.5 |  |  | | | 12 | 0.027 |  |  |  |  |  |  |  |  |  |  |  |  |  |

Life table for *An. stephensi* mosquitoes fed on blood containing different *P. berghei* ookinete densities in experiment 2. *N*(*t*), number of mosquitoes alive at the beginning of timepoint; *S*(*t*), Kaplan Meier survival function; *μ*(*t*), mortality rate mosquito^-1^ day^-1^.

|  | | |  |  | | | | |  |  | | |  |  | | |  |  | | |
| --- | --- | --- | --- | --- | --- | --- | --- | --- | --- | --- | --- | --- | --- | --- | --- | --- | --- | --- | --- | --- |
| **Ookinete density per μl of blood fed to mosquito group** | | |  | **0** | | | | |  | **100** | | |  | **400** | | |  | **2,000** | | |
|  | | |  |  | | |  |  |  |  |  |  |  |  |  |  |  |  |  |  |
| **Time post engorgement and midpoint (*t* ) (Days)** | | |  | ***N*(*t* )** | | | ***S*(*t* )** | ***μ*(*t* )** |  | ***N*(*t* )** | ***S*(*t* )** | ***μ*(*t* )** |  | ***N*(*t* )** | ***S*(*t* )** | ***μ*(*t* )** |  | ***N*(*t* )** | ***S*(*t* )** | ***μ*(*t* )** |
|  | |  |  | |  | |  |  |  |  |  |  |  |  |  |  |  |  |  |  |
| 0 | 0.5 |  |  | | 733 | | 1 | 0.001 |  | 565 | 1 | 0.002 |  | 662 | 1 | 0.003 |  | 502 | 1 | 0.008 |
| 1 | 1.5 |  |  | | 703 | | 0.999 | 0.010 |  | 539 | 0.998 | 0.024 |  | 632 | 0.997 | 0.044 |  | 473 | 0.992 | 0.063 |
| 2 | 2.5 |  |  | | 676 | | 0.989 | 0.015 |  | 506 | 0.974 | 0.020 |  | 584 | 0.953 | 0.038 |  | 423 | 0.929 | 0.043 |
| 3 | 3.5 |  |  | | 646 | | 0.974 | 0.014 |  | 476 | 0.955 | 0.004 |  | 541 | 0.917 | 0.017 |  | 384 | 0.890 | 0.031 |
| 4 | 4.5 |  |  | | 616 | | 0.960 | 0.015 |  | 454 | 0.951 | 0.018 |  | 512 | 0.902 | 0.008 |  | 352 | 0.862 | 0.023 |
| 5 | 5.5 |  |  | | 586 | | 0.946 | 0.015 |  | 426 | 0.934 | 0.007 |  | 488 | 0.895 | 0.012 |  | 324 | 0.842 | 0.019 |
| 6 | 6.5 |  |  | | 557 | | 0.932 | 0.011 |  | 403 | 0.928 | 0.005 |  | 462 | 0.884 | 0.030 |  | 298 | 0.827 | 0.007 |
| 7 | 7.5 |  |  | | 551 | | 0.922 | 0.013 |  | 401 | 0.923 | 0.007 |  | 448 | 0.857 | 0.013 |  | 295 | 0.821 | 0.020 |
| 8 | 8.5 |  |  | | 523 | | 0.910 | 0.011 |  | 378 | 0.916 | 0.013 |  | 422 | 0.845 | 0.009 |  | 269 | 0.804 | 0.004 |
| 9 | 9.5 |  |  | | 517 | | 0.900 | 0.008 |  | 373 | 0.904 | 0.003 |  | 418 | 0.837 | 0.002 |  | 268 | 0.801 | 0.007 |
| 10 | 10.5 |  |  | | 493 | | 0.893 | 0.018 |  | 352 | 0.902 | 0.009 |  | 397 | 0.835 | 0.010 |  | 246 | 0.795 | 0.028 |
| 11 | 11.5 |  |  | | 484 | | 0.876 | 0.008 |  | 349 | 0.894 | 0.000 |  | 393 | 0.827 | 0.008 |  | 239 | 0.773 | 0.017 |
| 12 | 12.5 |  |  | | 460 | | 0.869 | 0.013 |  | 329 | 0.894 | 0.003 |  | 369 | 0.821 | 0.014 |  | 215 | 0.760 | 0.014 |
| 13 | 13.5 |  |  | | 454 | | 0.858 | 0.013 |  | 328 | 0.891 | 0.003 |  | 364 | 0.810 | 0.005 |  | 212 | 0.749 | 0.009 |
| 14 | 14.5 |  |  | | 426 | | 0.847 | 0.007 |  | 307 | 0.888 | 0.013 |  | 342 | 0.805 | 0.009 |  | 190 | 0.742 | 0.021 |
| 15 | 15.5 |  |  | | 423 | | 0.841 | 0.019 |  | 303 | 0.877 | 0.017 |  | 339 | 0.798 | 0.012 |  | 186 | 0.727 | 0.005 |
| 16 | 16.5 |  |  | | 394 | | 0.825 | 0.013 |  | 278 | 0.862 | 0.004 |  | 314 | 0.789 | 0.019 |  | 165 | 0.723 | 0.018 |
| 17 | 17.5 |  |  | | 389 | | 0.814 | 0.018 |  | 277 | 0.859 | 0.004 |  | 308 | 0.774 | 0.013 |  | 162 | 0.709 | 0.012 |
| 18 | 18.5 |  |  | | 362 | | 0.800 | 0.011 |  | 256 | 0.856 | 0.012 |  | 284 | 0.763 | 0.018 |  | 140 | 0.701 | 0.000 |
| 19 | 19.5 |  |  | | 357 | | 0.791 | 0.011 |  | 253 | 0.846 | 0.012 |  | 279 | 0.750 | 0.018 |  | 140 | 0.701 | 0.014 |
| 20 | 20.5 |  |  | | 333 | | 0.782 | 0.012 |  | 230 | 0.836 | 0.013 |  | 254 | 0.737 | 0.024 |  | 118 | 0.691 | 0.017 |
| 21 | 21.5 |  |  | | 329 | | 0.772 | 0.024 |  | 227 | 0.825 | 0.018 |  | 248 | 0.719 | 0.024 |  | 116 | 0.679 | 0.017 |
| 22 | 22.5 |  |  | | 301 | | 0.754 | 0.013 |  | 202 | 0.811 | 0.010 |  | 222 | 0.702 | 0.014 |  | 94 | 0.667 | 0.021 |
| 23 | 23.5 |  |  | | 297 | | 0.744 | 0.017 |  | 200 | 0.803 | 0.045 |  | 219 | 0.692 | 0.032 |  | 92 | 0.653 | 0.033 |
| 24 | 24.5 |  |  | | 272 | | 0.731 | 0.029 |  | 171 | 0.766 | 0.000 |  | 192 | 0.670 | 0.021 |  | 69 | 0.632 | 0.043 |
| 25 | 25.5 |  |  | | 264 | | 0.710 | 0.019 |  | 171 | 0.766 | 0.029 |  | 188 | 0.656 | 0.016 |  | 66 | 0.604 | 0.045 |
| 26 | 26.5 |  |  | | | 239 | 0.696 | 0.029 |  | 146 | 0.744 | 0.034 |  | 165 | 0.646 | 0.030 |  | 43 | 0.577 | 0.023 |
| 27 | 27.5 |  |  | | | 232 | 0.676 | 0.026 |  | 141 | 0.719 | 0.007 |  | 160 | 0.626 | 0.031 |  | 42 | 0.563 | 0.048 |
| 28 | 28.5 |  |  | | | 199 | 0.658 | 0.040 |  | 119 | 0.713 | 0.000 |  | 135 | 0.607 | 0.044 |  | 19 | 0.537 | 0.158 |
| 29 | 29.5 |  |  | | | 191 | 0.632 | 0.042 |  | 119 | 0.713 | 0.017 |  | 128 | 0.580 | 0.023 |  | 16 | 0.452 |  |
| 30 | 30.5 |  |  | | | 160 | 0.605 | 0.050 |  | 97 | 0.702 | 0.041 |  | 103 | 0.566 | 0.029 |  |  |  |  |
| 31 | 31.5 |  |  | | | 151 | 0.575 | 0.073 |  | 93 | 0.673 | 0.043 |  | 98 | 0.550 | 0.051 |  |  |  |  |
| 32 | 32.5 |  |  | | | 119 | 0.533 | 0.076 |  | 67 | 0.644 | 0.030 |  | 70 | 0.522 | 0.057 |  |  |  |  |
| 33 | 33.5 |  |  | | | 88 | 0.493 | 0.068 |  | 44 | 0.624 | 0.023 |  | 44 | 0.492 | 0.023 |  |  |  |  |
| 34 | 34.5 |  |  | | | 61 | 0.459 |  |  | 22 | 0.610 |  |  | 22 | 0.481 |  |  |  |  |  |

Life table for *An. stephensi* mosquitoes fed on blood containing different *P. berghei* ookinete densities in experiment 3. *N*(*t*), number of mosquitoes alive at the beginning of timepoint; *S*(*t*), Kaplan Meier survival function; *μ*(*t*), mortality rate mosquito^-1^ day^-1^.

|  | | |  |  | | | | |  |  | | |  |  | | |  |  | | |
| --- | --- | --- | --- | --- | --- | --- | --- | --- | --- | --- | --- | --- | --- | --- | --- | --- | --- | --- | --- | --- |
| **Ookinete density per μl of blood fed to mosquito group** | | |  | **0** | | | | |  | **50** | | |  | **250** | | |  | **1,000** | | |
|  | | |  |  | | |  |  |  |  |  |  |  |  |  |  |  |  |  |  |
| **Time post engorgement and midpoint (*t* ) (Days)** | | |  | ***N*(*t* )** | | | ***S*(*t* )** | ***μ*(*t* )** |  | ***N*(*t* )** | ***S*(*t* )** | ***μ*(*t* )** |  | ***N*(*t* )** | ***S*(*t* )** | ***μ*(*t* )** |  | ***N*(*t* )** | ***S*(*t* )** | ***μ*(*t* )** |
|  | |  |  | |  | |  |  |  |  |  |  |  |  |  |  |  |  |  |  |
| 0 | 0.5 |  |  | | 625 | | 1 | 0.000 |  | 815 | 1 | 0.002 |  | 852 | 1 | 0.002 |  | 999 | 1 | 0.002 |
| 1 | 1.5 |  |  | | 602 | | 1.000 | 0.017 |  | 772 | 0.998 | 0.075 |  | 827 | 0.998 | 0.057 |  | 976 | 0.998 | 0.070 |
| 2 | 2.5 |  |  | | 571 | | 0.983 | 0.012 |  | 693 | 0.923 | 0.023 |  | 759 | 0.941 | 0.029 |  | 883 | 0.928 | 0.017 |
| 3 | 3.5 |  |  | | 542 | | 0.971 | 0.004 |  | 657 | 0.901 | 0.012 |  | 717 | 0.914 | 0.017 |  | 847 | 0.913 | 0.011 |
| 4 | 4.5 |  |  | | 520 | | 0.968 | 0.013 |  | 628 | 0.890 | 0.013 |  | 681 | 0.898 | 0.040 |  | 817 | 0.903 | 0.015 |
| 5 | 5.5 |  |  | | 492 | | 0.955 | 0.000 |  | 599 | 0.879 | 0.012 |  | 633 | 0.863 | 0.013 |  | 784 | 0.890 | 0.018 |
| 6 | 6.5 |  |  | | 472 | | 0.955 | 0.013 |  | 567 | 0.869 | 0.007 |  | 605 | 0.852 | 0.007 |  | 749 | 0.874 | 0.005 |
| 7 | 7.5 |  |  | | 465 | | 0.943 | 0.004 |  | 561 | 0.863 | 0.007 |  | 601 | 0.846 | 0.013 |  | 745 | 0.869 | 0.008 |
| 8 | 8.5 |  |  | | 443 | | 0.939 | 0.005 |  | 535 | 0.856 | 0.007 |  | 572 | 0.835 | 0.002 |  | 719 | 0.862 | 0.004 |
| 9 | 9.5 |  |  | | 441 | | 0.934 | 0.011 |  | 531 | 0.850 | 0.008 |  | 571 | 0.834 | 0.018 |  | 716 | 0.859 | 0.003 |
| 10 | 10.5 |  |  | | 415 | | 0.924 | 0.007 |  | 504 | 0.844 | 0.002 |  | 541 | 0.819 | 0.007 |  | 694 | 0.856 | 0.013 |
| 11 | 11.5 |  |  | | 412 | | 0.917 | 0.000 |  | 503 | 0.842 | 0.002 |  | 537 | 0.813 | 0.002 |  | 685 | 0.845 | 0.009 |
| 12 | 12.5 |  |  | | 391 | | 0.917 | 0.008 |  | 482 | 0.840 | 0.010 |  | 516 | 0.811 | 0.008 |  | 656 | 0.838 | 0.003 |
| 13 | 13.5 |  |  | | 388 | | 0.910 | 0.008 |  | 477 | 0.832 | 0.006 |  | 512 | 0.805 | 0.010 |  | 654 | 0.835 | 0.011 |
| 14 | 14.5 |  |  | | 364 | | 0.903 | 0.011 |  | 454 | 0.826 | 0.007 |  | 487 | 0.797 | 0.004 |  | 626 | 0.826 | 0.006 |
| 15 | 15.5 |  |  | | 358 | | 0.893 | 0.000 |  | 451 | 0.821 | 0.007 |  | 485 | 0.794 | 0.014 |  | 622 | 0.821 | 0.014 |
| 16 | 16.5 |  |  | | 338 | | 0.893 | 0.003 |  | 427 | 0.815 | 0.009 |  | 458 | 0.782 | 0.011 |  | 593 | 0.809 | 0.008 |
| 17 | 17.5 |  |  | | 337 | | 0.890 | 0.003 |  | 423 | 0.808 | 0.005 |  | 453 | 0.774 | 0.029 |  | 588 | 0.802 | 0.012 |
| 18 | 18.5 |  |  | | 316 | | 0.888 | 0.000 |  | 401 | 0.804 | 0.002 |  | 419 | 0.752 | 0.005 |  | 561 | 0.793 | 0.016 |
| 19 | 19.5 |  |  | | 316 | | 0.888 | 0.006 |  | 400 | 0.802 | 0.025 |  | 417 | 0.748 | 0.002 |  | 546 | 0.780 | 0.024 |
| 20 | 20.5 |  |  | | 294 | | 0.882 | 0.010 |  | 370 | 0.782 | 0.022 |  | 396 | 0.746 | 0.010 |  | 512 | 0.761 | 0.018 |
| 21 | 21.5 |  |  | | 291 | | 0.873 | 0.000 |  | 361 | 0.765 | 0.008 |  | 391 | 0.739 | 0.015 |  | 502 | 0.748 | 0.018 |
| 22 | 22.5 |  |  | | 271 | | 0.873 | 0.004 |  | 338 | 0.759 | 0.012 |  | 365 | 0.727 | 0.014 |  | 473 | 0.735 | 0.019 |
| 23 | 23.5 |  |  | | 270 | | 0.870 | 0.007 |  | 334 | 0.750 | 0.018 |  | 360 | 0.717 | 0.011 |  | 464 | 0.721 | 0.022 |
| 24 | 24.5 |  |  | | 248 | | 0.863 | 0.016 |  | 308 | 0.736 | 0.023 |  | 336 | 0.710 | 0.018 |  | 431 | 0.705 | 0.021 |
| 25 | 25.5 |  |  | | 244 | | 0.850 | 0.000 |  | 301 | 0.719 | 0.023 |  | 330 | 0.697 | 0.015 |  | 421 | 0.690 | 0.024 |
| 26 | 26.5 |  |  | | | 224 | 0.850 | 0.009 |  | 273 | 0.703 | 0.026 |  | 303 | 0.686 | 0.023 |  | 383 | 0.674 | 0.042 |
| 27 | 27.5 |  |  | | | 222 | 0.842 | 0.014 |  | 266 | 0.685 | 0.030 |  | 291 | 0.670 | 0.021 |  | 367 | 0.646 | 0.030 |
| 28 | 28.5 |  |  | | | 199 | 0.831 | 0.005 |  | 238 | 0.664 | 0.021 |  | 264 | 0.657 | 0.008 |  | 336 | 0.626 | 0.033 |
| 29 | 29.5 |  |  | | | 198 | 0.826 | 0.005 |  | 233 | 0.650 | 0.017 |  | 262 | 0.652 | 0.027 |  | 325 | 0.606 | 0.034 |
| 30 | 30.5 |  |  | | | 176 | 0.822 | 0.006 |  | 208 | 0.639 | 0.043 |  | 234 | 0.634 | 0.038 |  | 290 | 0.585 | 0.072 |
| 31 | 31.5 |  |  | | | 175 | 0.818 | 0.046 |  | 199 | 0.611 | 0.040 |  | 225 | 0.610 | 0.018 |  | 267 | 0.543 | 0.041 |
| 32 | 32.5 |  |  | | | 147 | 0.780 | 0.014 |  | 171 | 0.587 | 0.035 |  | 201 | 0.599 | 0.040 |  | 236 | 0.521 | 0.038 |
| 33 | 33.5 |  |  | | | 144 | 0.770 | 0.021 |  | 163 | 0.566 | 0.031 |  | 192 | 0.575 | 0.068 |  | 226 | 0.501 | 0.080 |
| 34 | 34.5 |  |  | | | 121 | 0.754 | 0.017 |  | 127 | 0.549 | 0.087 |  | 158 | 0.536 | 0.051 |  | 184 | 0.461 | 0.076 |
| 35 | 35.5 |  |  | | | 119 | 0.741 | 0.008 |  | 116 | 0.501 | 0.078 |  | 148 | 0.509 | 0.054 |  | 169 | 0.426 | 0.071 |
| 36 | 36.5 |  |  | | | 98 | 0.735 | 0.020 |  | 85 | 0.462 | 0.059 |  | 118 | 0.482 | 0.042 |  | 136 | 0.396 | 0.074 |
| 37 | 37.5 |  |  | | | 96 | 0.720 | 0.042 |  | 80 | 0.435 | 0.050 |  | 111 | 0.461 | 0.090 |  | 124 | 0.367 | 0.081 |
| 38 | 38.5 |  |  | | | 72 | 0.690 | 0.042 |  | 53 | 0.413 | 0.094 |  | 79 | 0.420 | 0.051 |  | 89 | 0.337 | 0.101 |
| 39 | 39.5 |  |  | | | 69 | 0.661 | 0.058 |  | 47 | 0.374 | 0.106 |  | 74 | 0.398 | 0.081 |  | 79 | 0.303 | 0.139 |
| 40 | 40.5 |  |  | | | 41 | 0.623 | 0.098 |  | 19 | 0.335 | 0.053 |  | 48 | 0.366 | 0.042 |  | 46 | 0.261 | 0.065 |
| 41 | 41.5 |  |  | | | 36 | 0.562 |  |  | 15 | 0.317 |  |  | 46 | 0.351 |  |  | 42 | 0.244 |  |
