# Supplementary material for: Anopheles mortality is both age- and Plasmodium-density dependent: implications for malaria transmission
Source: Malar J. 2009 Oct 12;8:228. doi: 10.1186/1475-2875-8-228 (PMC2770541; doi:10.1186/1475-2875-8-228)
Supplement: Additional file 5 — Mosquito life expectancy with time post-engorgement and mean number of oocysts on day 10 post-engorgement. Graph illustrating how mosquito life expectancy depends upon time post-engorgement and the mean number of oocysts per mosquito 10 days post bloodfeed. [file 1475-2875-8-228-S5.DOC]

**Additional file 5: Mosquito life expectancy with time post-engorgement and mean number of oocysts on day 10 post-engorgement.**

**
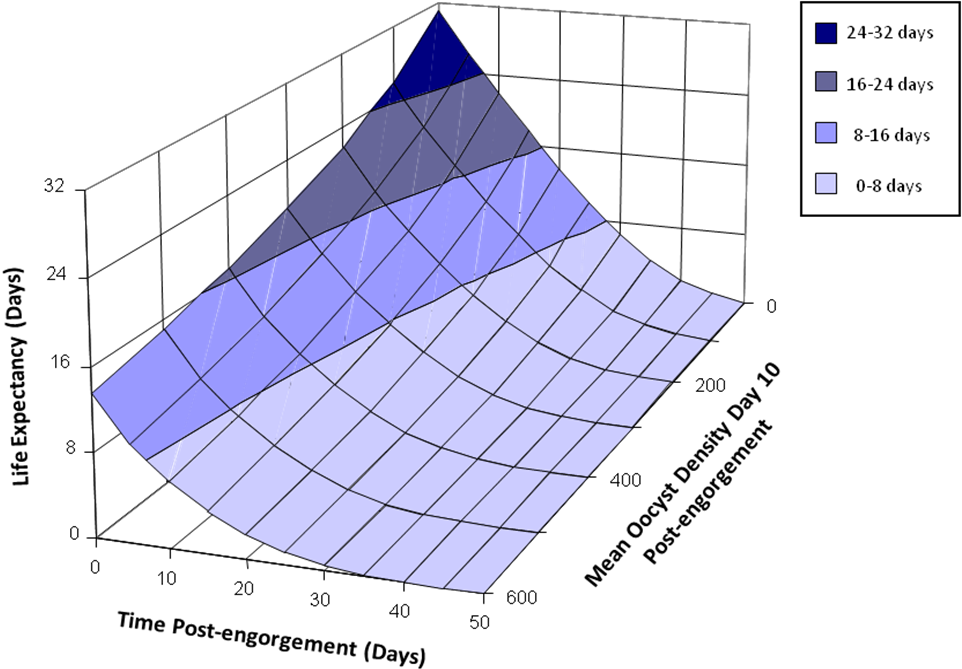
**

Life expectancy of mosquitoes maintained in the laboratory, plotted against time post-engorgement and the mean number of oocysts per mosquito 10 days post bloodfeed (shown in Table 1 and [44]). The parameters relating parasite density on day 10 to the mortality rate experienced by mosquitoes were calculated and incorporated into the expression for life expectancy as described in additional file 3: ‘Calculating how life-expectancy of mosquitoes varies with mean oocyst density on day 10and time post-engorgement’. Parameter values: *a_0_* = 1.37x10^-4^, *a_1_* = 1.91x10^-7^, *b_0_* = -3.76x10^-3^, *b_1_* = -2.79x10^-6^, *c_0_* = 3.21x10^-2^, *c_1_* = 9.13x10^-5^.
